# Evaluation of the association of area-level socioeconomic deprivation and breast cancer recurrence by oestrogen receptor subtypes in Scotland

**DOI:** 10.1186/s13058-023-01704-6

**Published:** 2023-10-03

**Authors:** Hayley M. Dunlop, Linda J. Williams, Peter S. Hall, Matthew Barber, Christine Dodds, Jonine D. Figueroa

**Affiliations:** 1 The Ohio State University College of Medicine, Columbus, OH USA; 2 Edinburgh Clinical Trials Unit, The Usher Institute, The University of Edinburgh, Edinburgh, EH16 4UX UK; 3 Institute of Genetics and Cancer, University of Edinburgh, Edinburgh, UK; 4 Edinburgh Breast Unit, NHS Lothian, Western General Hospital, Edinburgh, UK; 5 Southeast Scotland Cancer Network (SCAN), Edinburgh, UK; 6 Centre for Global Health, The Usher Institute, The University of Edinburgh, Edinburgh, EH8 9AG UK; 7 Division of Cancer Epidemiology and Genetics, National Cancer Institute, National Institutes of Health, Bethesda, MD USA

**Keywords:** Breast cancer, Recurrence, Disparities

## Abstract

**Background:**

Women from socioeconomically deprived areas have lower breast cancer (BC) incidence rates for screen-detected oestrogen receptor (ER) + tumours and higher mortality for select tumour subtypes. We aimed to determine if ipsilateral breast cancer recurrence (IBR) differs by Scottish Index of Multiple Deprivation (SIMD) quintile and tumour subtype in Scotland.

**Methods:**

Patient data for primary invasive BC diagnosed in 2007–2008 in Scotland was analysed. Manual case-note review for 3495 patients from 10 years post-diagnosis was used. To determine the probability of IBR while accounting for the competing risk of death from any cause, cumulative incidence functions stratified by ER subtype and surgery were plotted. Multivariable Cox Proportional Hazards models were used to estimate the association of SIMD accounting for other predictors of IBR.

**Results:**

Among 2819 ER + tumours, 423 patients had a recurrence and 438 died. SIMD was related to death (*p *= 0.018) with the most deprived more likely to have died in the 10-year period (17.7% vs. 12.9%). We found no significant differences by SIMD in prognostic tumour characteristics (grade, TNM stage, treatment, screen-detection) or risk of IBR. Among 676 patients diagnosed with ER- tumours, 105 died and 185 had a recurrence. We found no significant differences in prognostic tumour characteristics by SIMD except screen detection with the most deprived more likely than the least to have their tumours detected from screening (46.9% vs. 28%, *p *= 0.03). Among patients with ER- tumours, 50% had mastectomy and the most deprived had increased 5-year IBR risk compared to the least deprived (HR 3.03 [1.41–6.53]).

**Conclusions:**

IBR is not a major contributor to mortality differences by SIMD for the majority of BC patients in our study. The lack of inequities in IBR are likely due to standardised treatment protocols and access to healthcare. The association with socioeconomic deprivation and recurrence for ER- tumours requires further study.

**Supplementary Information:**

The online version contains supplementary material available at 10.1186/s13058-023-01704-6.

## Introduction

Female breast cancer (BC) is the most common cancer worldwide regardless of sex [1] and in Scotland accounts for 28.8% of cancer cases [2]. BC is comprised of multiple molecular subtypes, each of which have their own prognosis, treatment, and aetiology [3–13]. Socio-economic disparities in BC incidence and survival have been described in Scotland [6, 14] and multiple countries around the world [15–18]. We previously reported that women in Scotland from socioeconomically deprived areas have lower breast cancer (BC) incidence rates for screen-detected oestrogen receptor (ER) + tumours and higher mortality for HER2-enriched and Luminal B subtypes [6].

In this study, we aimed to determine if there were differences in local BC recurrence by SIMD quintile and ER subtype. To investigate this, we utilised a dataset with > 4000 Scottish women diagnosed with BC from 2007–2008, all of whom received treatment through Scotland’s National Health Service (NHS), which provides universal healthcare. Given that local and regional recurrences influence the occurrence of distant metastases and subsequent BC death [19, 20], examining differences in recurrence by SES and ER subtype could inform if inequities in outcomes exist and if interventions to eliminate these disparities are needed.

## Materials and methods

### Study population

#### Data and cohort definition

We used data from a subset of patients with a primary breast tumour diagnosed in 2007–2008. At this time, recurrence data was not routinely recorded and there was uncertainty as to whether such data could be collected. Finding data relating to individual patients with recurrence is relatively easy, with access to electronic records and an efficient process to refer patients back to the multidisciplinary tumour board (MDT) in the event of any breast issues following treatment. To verify each patient's status, all records were checked and updated using national and local electronic health care data sources e.g. Scottish Care Information (SCI Store). Invitation to participate in this audit was extended to all NHS Scotland health boards and involved detailed follow-up by manual case-note review from 10 years post-diagnosis. Cancer deaths were ascertained through passive data collection by the Scottish Cancer Registry.

A fully anonymised dataset was compiled from the audit database specifically to examine BC recurrence. This dataset included 4097 Scottish women diagnosed with a primary breast tumour and Scottish Index of Multiple Deprivation (SIMD) quintile available. SIMD is a relative measure of socioeconomic deprivation between 6.976 small areas (data zones) in Scotland, with 700–800 people per data zone [21]. This index incorporates multiple elements of deprivation, including income, education, employment, health, crime, access to services, and housing, and quintiles are calculated at the country level [20]. The postcode where patients were resident at the time of diagnosis was used to determine their SIMD quintile for this study. During our study period, SIMD was recorded with SIMD 1 as the least deprived and SIMD 5 as the most deprived [22].

**Fig. 1:**
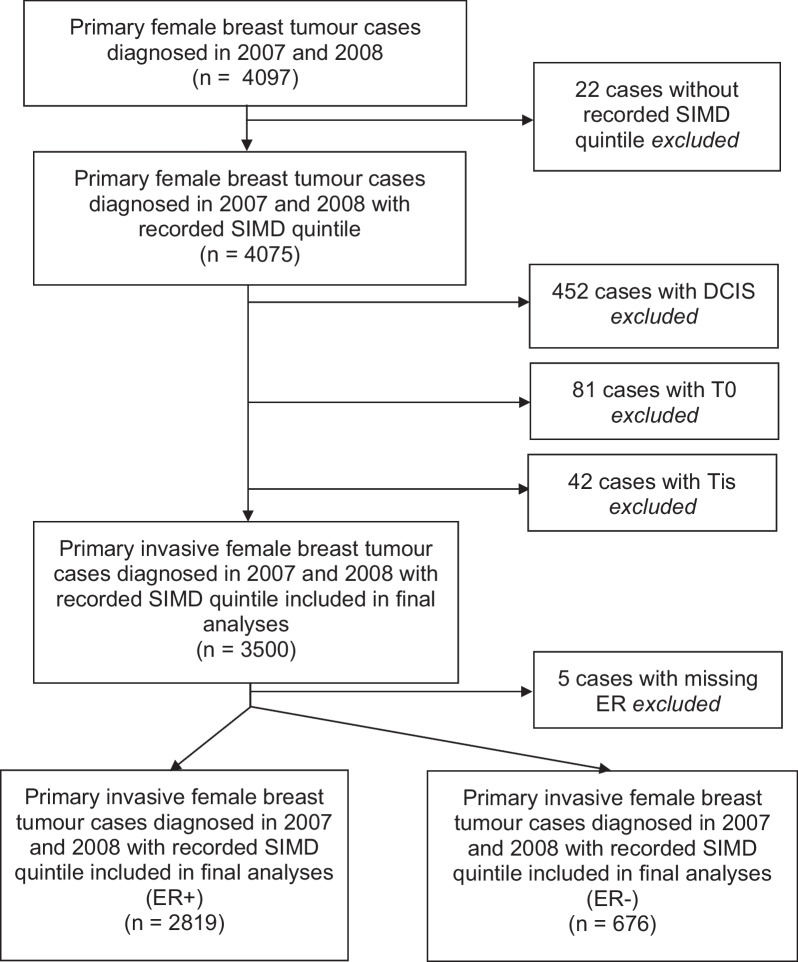
Establishing the cohorts for breast cancer recurrence analyses

We excluded women with non-invasive diagnoses due to possible different aetiology of *In-situ* cases (N = 597) and missing ER status (N = 5), hence 3,495 women were available for analysis (Fig. 1) [23]. Of the cases clinically diagnosed as Tis, 42 were noted to be invasive breast cancers after surgical resection. These cases were excluded from the final cohort as pathologic T stage was unavailable.

A three-level categorical variable of age was used in analysis: < 50 (prior to routine screening invitation), 50–70 (period of routine screening invitation), > 70 (after period of routine screening invitation, but patients encouraged to attend) [24]. Regional health board where care was sought was obtained and recorded as one of the three cancer networks (West of Scotland Cancer Network, Southeast Scotland Cancer Network, North of Scotland Cancer Network). Tumour grade was defined as grade 1 (well differentiated), grade 2 (moderately differentiated), or grade 3 (poorly differentiated). Information on method of detection was used to categorize each diagnosed primary tumour as screen- detected, not screen-detected (which included all remaining options: clinical examination, incidental finding, self-referral, etc.) and unknown [25]. Clinical TNM stage at diagnosis was derived from individual T, N and M clinical stages. Treatment data including surgery (breast-conserving surgery, mastectomy), chemotherapy, and radiotherapy were also included in analyses. Endocrine therapy was excluded from the analyses to avoid issues of multicollinearity, as > 97% of patients in the study cohort with ER+ cancers received standard of care treatment with endocrine therapy (data not shown).

### Statistical analysis

The primary outcome of interest was time to ipsilateral breast cancer recurrence (IBR) defined as an additional BC in the ipsilateral breast or chest wall diagnosed after initial treatment. Death was considered a competing risk for IBR and cause of death (alive, BC death, other cancer death, non-cancer death, unknown, missing) was included. The primary study endpoint was any IBR. Our null hypothesis was that there was no difference between SIMD and IBR and this did not differ by ER status.

To assess the probability of IBR while accounting for the competing risk of death from any cause, competing risk analyses were performed using cumulative incidence functions (CIFs). The event of interest in these analyses was the probability of experiencing IBR in the first 10 years after diagnosis in the presence of the competing risk of death. All time-to-event curves were right-censored at 10 years. The test for equality was used to assess whether there was a significant difference in the primary or secondary outcomes by SIMD.

Hazard rate curves for time to recurrence were smoothed with an Epanechinikov kernel to determine if the risk of IBR varied between SIMD quintiles at different time points when stratified by surgery and ER status [26].

Multivariable Cox proportional hazards models were used to estimate hazards ratios (HR) the association with SIMD accounting for other predictors of IBR as well as cancer network. Due to proportional hazards violation, Cox models were censored to 5 years follow-up time. Landmark analyses were performed for ER+ cancers to examine IBR for all patients who survived to 5 years after diagnosis to examine the risk of recurrence between 5 and 10 years. These could not be performed for ER- cancers as there were insufficient data to analyse the period between five and 10 years.

There are no missing data for the exposure variable (SIMD quintile) or for the outcome variable of IBR. Further discussion of variable definitions, missing data, and choice of methods are included in Additional file 1: Methods. All analyses were conducted in R Studio Version 1.3.1093. No adjustments for multiple testing have been made.

## Results

### Descriptive characteristics of the population

Of the 3495 women diagnosed with an invasive BC, 27.1% were in SIMD 1 (least deprived), and (10.2%) were in SIMD 5 (most deprived). Approximately half of cases were from SCAN and approximately a quarter of cases from WOSCAN and NOSCAN.

The majority of tumours were ER+ (N = 2819, 80%) (Fig. 1). ER+ tumour diagnoses were highest among the least deprived quintile (SIMD 1, 27.2%) and lowest in the most deprived quintile (SIMD 5, 10.4% see Table 1). Tumours were mostly Grade 2 (53.8%) or Grade 3 (28.0%) (Table 1). Most ER+ tumours were low stage (Stage I 53.4% or Stage II 38.7%). SIMD was not significantly associated with any tumour characteristics, however, a greater proportion of those in the most deprived quintile (SIMD 5 = 33.3%) had Grade 3 tumours than those in other SIMD quintiles (Table 1). A statistically significant trend in event types (dead, no IBR; alive, no IBR; IBR) was observed, with a greater proportion of those in the most deprived SIMD quintile (17.7%) having a death without IBR and 15.6% having an IBR (Table 1). For ER+ tumours, no trend by SIMD for mode of detection was observed for those in the 50–70 age group who are eligible for screening on the NHS (Table 1). For nearly all SIMD quintiles, ~ 60% of tumours were screen detected within the 50–70 age group.

**Table 1:** Descriptive table of 2819 ER-positive breast cancer cases by Scottish Index for Multiple Deprivation (SIMD) diagnosed in Scotland from 2007–2008

Variable	Categories of variable	Total Patients (N = 2819) (Column %)	SIMD 1 (least deprived) (N = 767)	SIMD 2 (N = 661)	SIMD 3 (N = 601)	SIMD 4 (N = 496)	SIMD 5 (most deprived) (N = 294)
Event type	Dead, no recurrence	438 (15.5%)	99 (12.9%)	107 (16.2%)	91 (15.1%)	89 (17.9%)	52 (17.7%)
	Alive, no recurrence	1958 (69.5%)	547 (71.3%)	456 (69.0%)	413 (68.7%)	346 (69.8%)	196 (66.7%)
	Recurrence (+/− death following recurrence)	423 (15.0%)	121 (15.8%)	98 (14.8%)	97 (16.1%)	61 (12.3%)	46 (15.6%)
Cause of death^a^	Breast cancer	249 (36.2%)	70 (40.5%)	48 (31.8%)	49 (34.8%)	44 (33.3%)	38 (41.8%)
	Non breast cancer death	319 (46.4%)	76 (43.9%)	65 (43.0%)	59 (41.8%)	73 (55.3%)	46 (50.5%)
Tumour grade	Grade 1	483 (17.1%)	134 (17.5%)	105 (15.9%)	91 (15.1%)	96 (19.4%)	57 (19.4%)
	Grade 2	1516 (53.8%)	424 (55.3%)	360 (54.5%)	333 (55.4%)	264 (53.2%)	135 (45.9%)
	Grade 3	788 (28.0%)	201 (26.2%)	187 (28.3%)	170 (28.3%)	133 (26.8%)	97 (33.0%)
TNM stage	I	1088 (53.4%)	316 (55.0%)	235 (53.4%)	221 (54.8%)	206 (53.2%)	110 (47.0%)
	II	790 (38.7%)	211 (36.7%)	177 (40.2%)	143 (35.5%)	151 (39.0%)	108 (46.2%)
	III	161 (7.9%)	48 (8.3%)	268 (6.4%)	39 (9.7%)	30 (7.8%)	16 (6.8%)
Age at diagnosis	< 50	580 (20.6%)	147 (19.2%)	132 (20.0%)	142 (23.6%)	102 (20.6%)	57 (19.4%)
	50–70	1603 (56.9%)	442 (57.6%)	374 (56.6%)	343 (57.1%)	276 (55.6%)	168 (57.1%)
	70 +	636 (22.6%)	178 (23.2%)	155 (23.4%)	116 (19.3%)	118 (23.8%)	69 (23.5%)
Treatment received	Breast-conserving surgery	1726 (61.2%)	498 (64.9%)	395 (59.8%)	366 (60.9%)	288 (58.1%)	179 (60.9%)
	Mastectomy	1093 (38.8%)	269 (35.1%)	266 (40.2%)	235 (39.1%)	208 (41.9%)	115 (39.1%)
	Chemotherapy	1087 (38.6%)	291 (38.0%)	248 (37.6%)	238 (39.7%)	187 (37.8%)	123 (41.8%)
	Radiotherapy	2089 (74.4%)	584 (76.3%)	462 (70.0%)	450 (75.4%)	369 (74.7%)	224 (76.5%)
Screening^b^	Screen detected	969 (60.4%)	262 (59.3%)	232 (62.0%)	215 (62.7%)	162 (58.7%)	98 (58.3%)
	Not screen detected	634 (39.6%)	180 (40.7%)	388 (38.0%)	366 (37.3%)	301 (41.3%)	189 (41.7%)

^a^Other cancer death and non-cancer death collapsed into category “non breast cancer death”

^b^Restricted to 50–70 age group (screening-eligible on the NHS)

Of the 676 ER- cases, approximately half were in the least deprived quintiles (SIMD 1 = 26.7%, SIMD 2 = 25.4%) with the remainder in the intermediate deprivation quintiles (SIMD 3 = 21.4%and SIMD 4 = 16.9%, and the most deprived quintile (SIMD 5 = 9.5%) (Table 2). A greater proportion of tumours were Grade 3 (83.9%) than Grade 1 or 2 (16.1%) (Table 2). Most women with ER-tumours were diagnosed at Stage II (55.4%) followed by Stage I (32.7%) and then Stage III (12.0%, Table 2). There was no significant association with SIMD by tumour or event outcomes except for mode of detection, with a greater proportion of those aged 50–70 in the most deprived quintile having screen detected ER- tumours (SIMD 5 = 46.9%) than those aged 50–70 in less deprived categories (SIMD 1 = 28.0%) (Table 2).

**Table 2:** Descriptive table of 676 ER-negative breast cancer cases by Scottish index for multiple deprivation (SIMD) diagnosed in Scotland from 2007–2008

Variable	Categories of variable	Total Patients (N = 676) (Column %)	SIMD 1 (least deprived) (N = 181)	SIMD 2 (N = 172)	SIMD 3 (N = 145)	SIMD 4 (N = 114)	SIMD 5 (most deprived) (N = 64)
Event type	Dead, no recurrence	105 (15.5%)	24 (13.3%)	28 (16.3%)	21 (14.5%)	23 (20.2%)	9 (14.1%)
	Alive, no recurrence	386 (57.1%)	118 (65.2%)	98 (57.0%)	73 (50.3%)	64 (56.1%)	33 (51.6%)
	Recurrence (+/− death following recurrence)	185 (27.4%)	39 (21.5%)	46 (26.7%)	51 (35.2%)	27 (23.7%)	22 (34.4%)
Cause of death^a^	Breast cancer	116 (46.8%)	24 (44.4%)	27 (43.5%)	34 (55.7%)	18 (40.9%)	14 (48.1%)
	Non breast cancer death	61 (24.6%)	14 (25.9%)	15 (24.2%)	10 (16.4%)	16 (36.4%)	6 (22.2%)
Tumour grade	Grade 1/2	105 (16.1%)	32 (18.6%)	22 (13.4%)	20 (14.3%)	23 (20.4%)	8 (12.5%)
	Grade 3	548 (83.9%)	140 (81.4%)	142 (86.6%)	120 (85.7%)	90 (79.6%)	56 (87.5%)
TNM stage	I	131 (32.7%)	34 (34.7%)	33 (33.0%)	21 (23.6%)	23 (32.4%)	20 (46.5%)
	II/III	270 (67.3%)	64 (65.3%)	67 (67.0%)	68 (76.4%)	48 (67.6%)	23 (53.5%)
Age at diagnosis	< 50	186 (27.5%)	46 (25.4%)	46 (26.7%)	44 (30.3%)	32 (28.1%)	18 (28.1%)
	50–70	348 (51.5%)	100 (55.2%)	91 (52.9%)	71 (49.0%)	54 (47.4%)	32 (50.0%)
	70 +	142 (21.0%)	35 (19.3%)	35 (20.3%)	30 (20.7%)	28 (24.6%)	14 (21.9%)
Treatment received	Breast-conserving surgery	335 (49.6%)	91 (50.3%)	83 (48.3%)	69 (47.6%)	54 (47.4%)	38 (59.4%)
	Mastectomy	341 (50.4%)	90 (49.7%)	89 (51.7%)	76 (52.4%)	60 (52.6%)	26 (40.6%)
	Chemotherapy	513 (76.0%)	137 (75.7%)	132 (77.2%)	108 (74.5%)	86 (75.4%)	50 (78.1%)
	Radiotherapy	492 (73.2%)	134 (74.4%)	126 (73.7%)	103 (71.0%)	82 (73.2%)	47 (73.4%)
Screening^b^	Screen detected	114 (32.8%)	28 (28.0%)	26 (28.6%)	26 (36.6%)	19 (35.2%)	15 (46.9%)
	Not screen detected	234 (67.2%)	72 (72.0%)	65 (71.4%)	45 (63.4%)	35 (64.8%)	17 (53.1%)

^a^Other cancer death and non-cancer death collapsed into category “non breast cancer death”

^b^Restricted to 50–70 age group (screening-eligible on the NHS)

### IBR risk by deprivation stratum: cumulative incidence functions by ER status

**Fig. 2:**
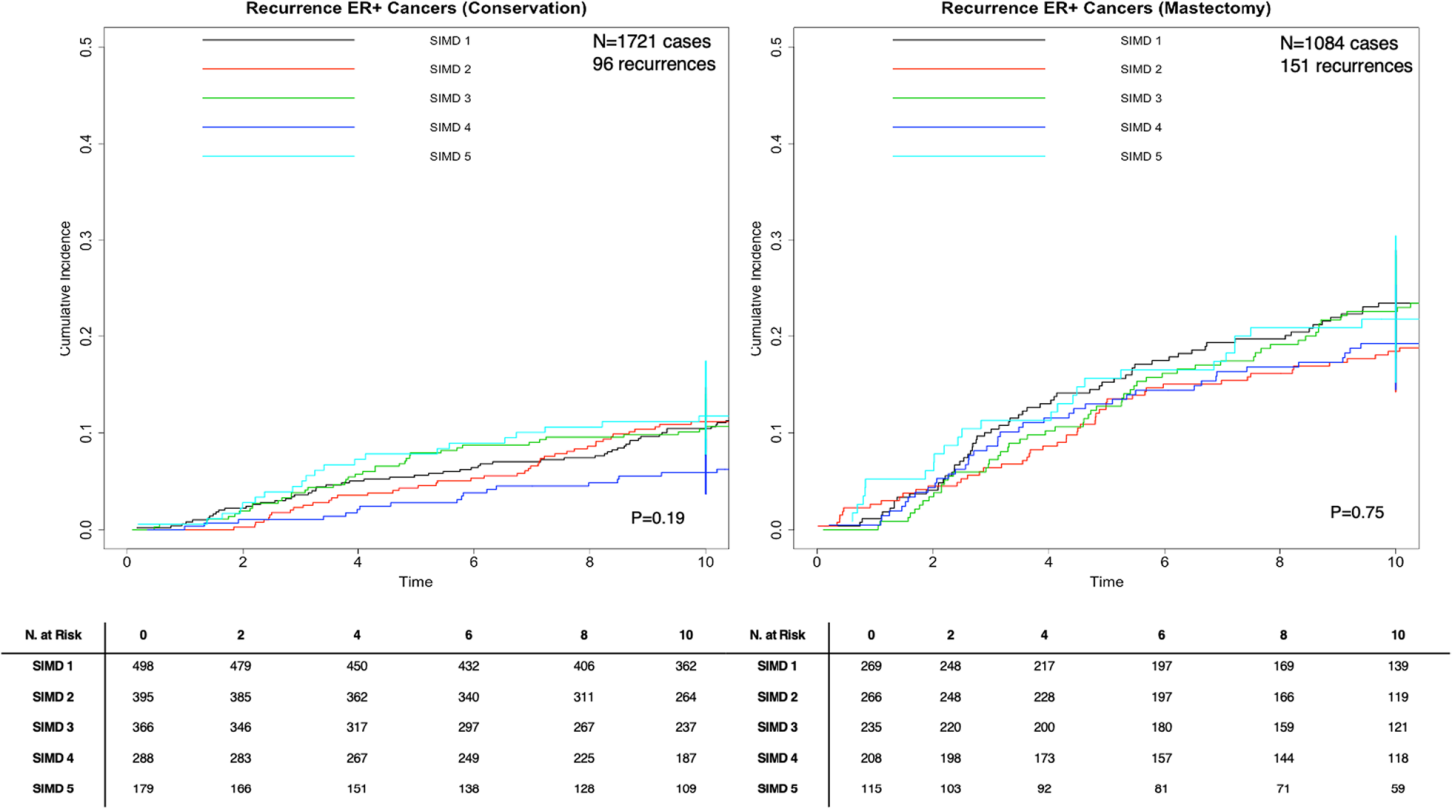
Cumulative incidence function stratified by surgery for 2819 ER+ breast cancer patients diagnosed in Scotland in 2007–2008 with 10 year recurrence data, *P* value test for equality across groups. ^1^SIMD 1 represents the most affluent area. SIMD 5 represents the most deprived area

**Fig. 3:**
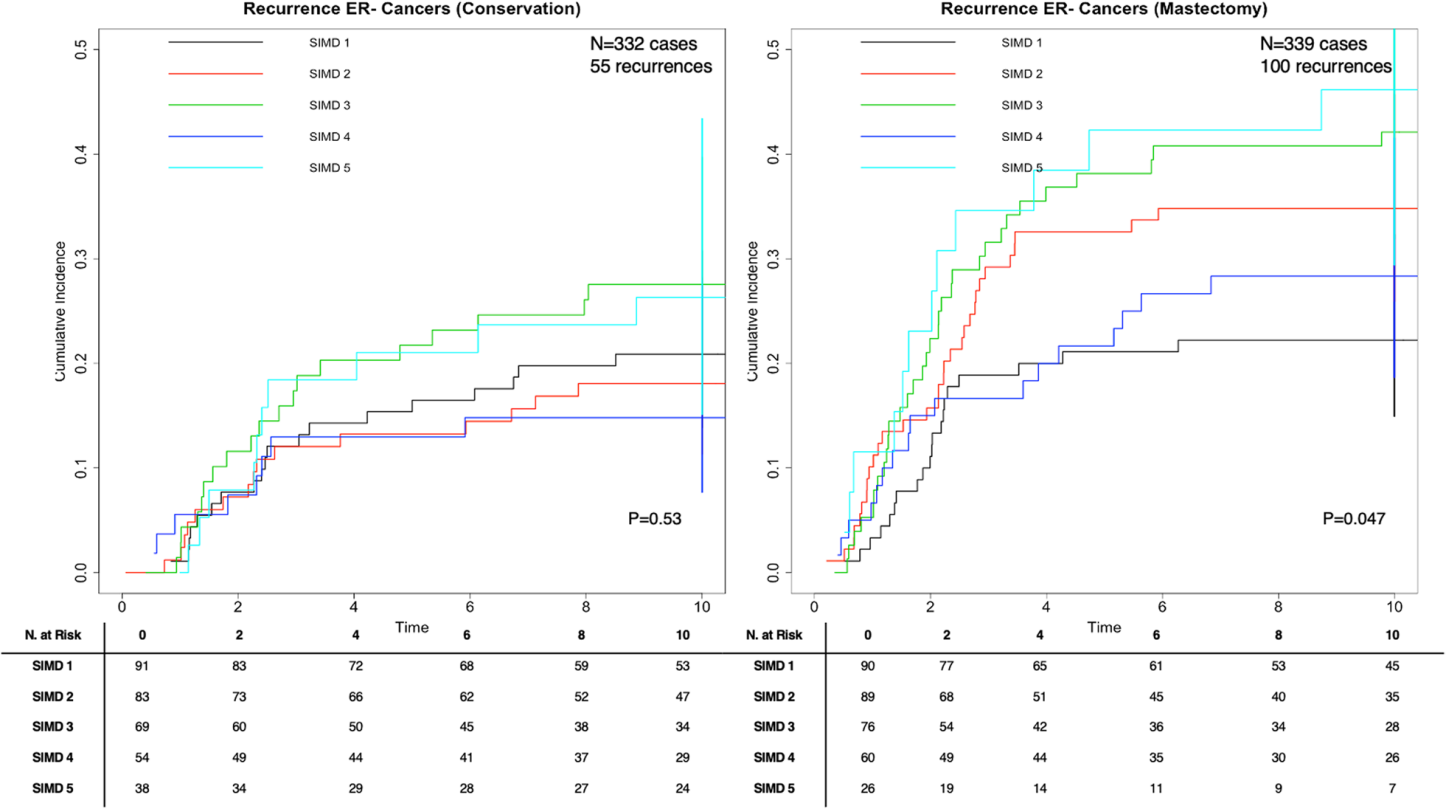
Cumulative incidence function stratified by surgery for 676 ER- breast cancer patients diagnosed in Scotland in 2007–2008 with 10 year recurrence data, *P* value test for equality across groups. ^1^SIMD 1 represents the most affluent area. SIMD 5 represents the most deprived area

CIF was performed to assess if differences in 10-year IBR exist by SIMD quintile stratified by breast surgery (breast conservation vs. mastectomy) and ER status in the presence of the competing risk of death from any cause. For ER+ tumours, no significant difference in risk of IBR by SIMD quintile for the breast conservation group (*p *= 0.19) or the mastectomy group (the p-value test for equality = 0.75) (Fig. 2). For ER- tumours, the p-value test for equality across groups showed a statistically significant difference in risk of IBR by SIMD quintile (*p *= 0.047) in the mastectomy group, but not for the breast conservation group (Fig. 3). In the mastectomy group, the probability of IBR for the least deprived (SIMD 1) was 0.32 (95% CI 0.15, 0.32), 0.35 (0.26, 0.46) for SIMD 2, 0.35 (0.28, 0.44) for SIMD 3, 0.28 (0.19, 0.42) for SIMD 4, and 0.46 (0.29, 0.67) for the most deprived (SIMD 5).

### Hazard rate curves by deprivation stratum by ER status

**Fig. 4:**
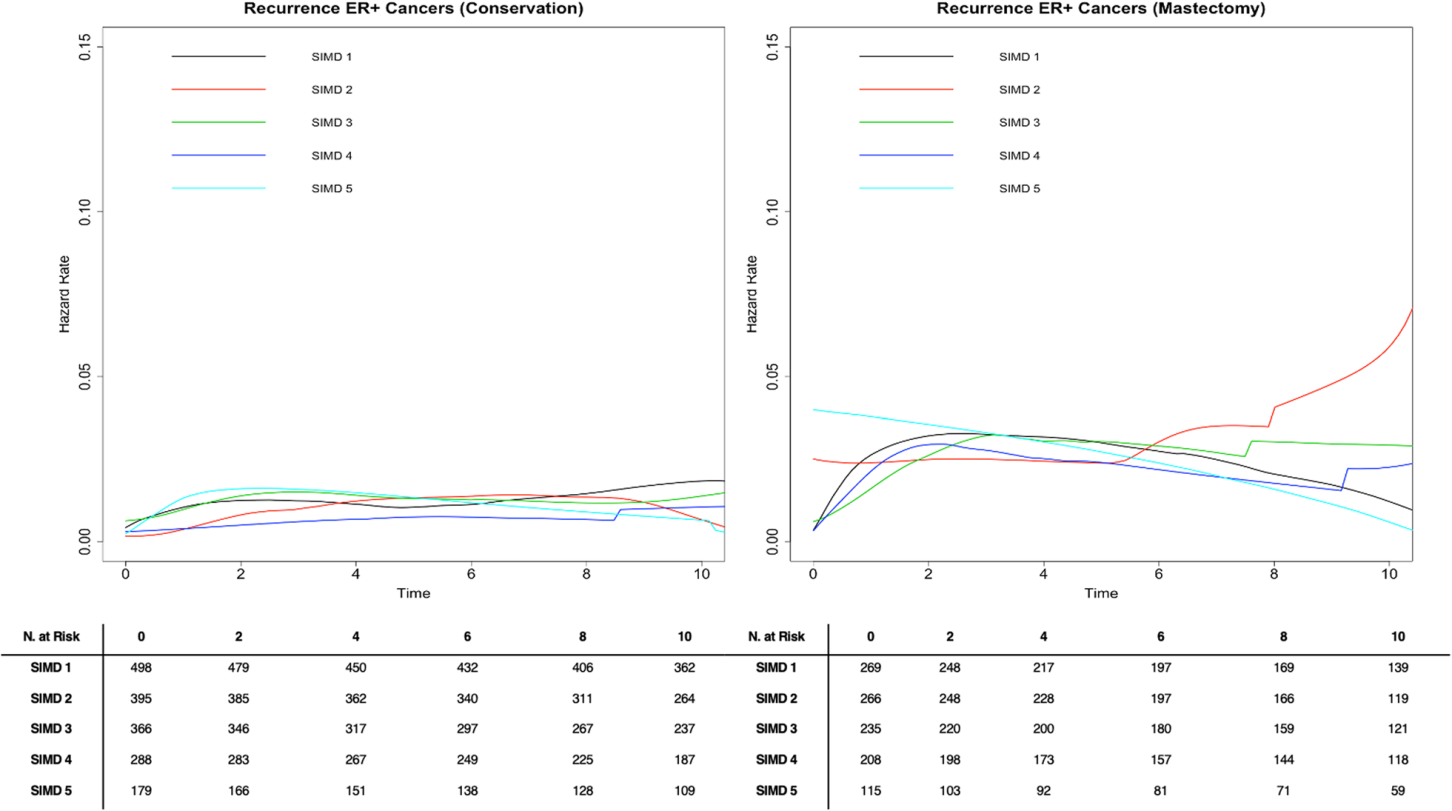
Epanechnikov kernel-smoothed hazard function curve for breast cancer recurrence stratified by surgery for 2819 ER+ breast cancer patients diagnosed in Scotland in 2007–2008 with 10 year follow-up data. ^1^SIMD 1 represents the most affluent area. SIMD 5 represents the most deprived area

To determine if the risk of IBR at a given time point varied over time between SIMD quintiles, kernel-smoothed hazard rate curves were plotted. For patients with ER+ tumours who underwent breast conserving surgery, there was no evidence of variability in hazard rates between deprivation strata at any time point, and these hazard rates remained fairly constant over time (Fig. 4). For patients with ER+ tumours who underwent mastectomy, those in the most deprived SIMD quintile had a slightly higher hazard rate in the first 2 years after diagnosis when compared to the other SIMD quintiles, but these hazard rates converged at 2 years (Fig. 4).

**Fig. 5:**
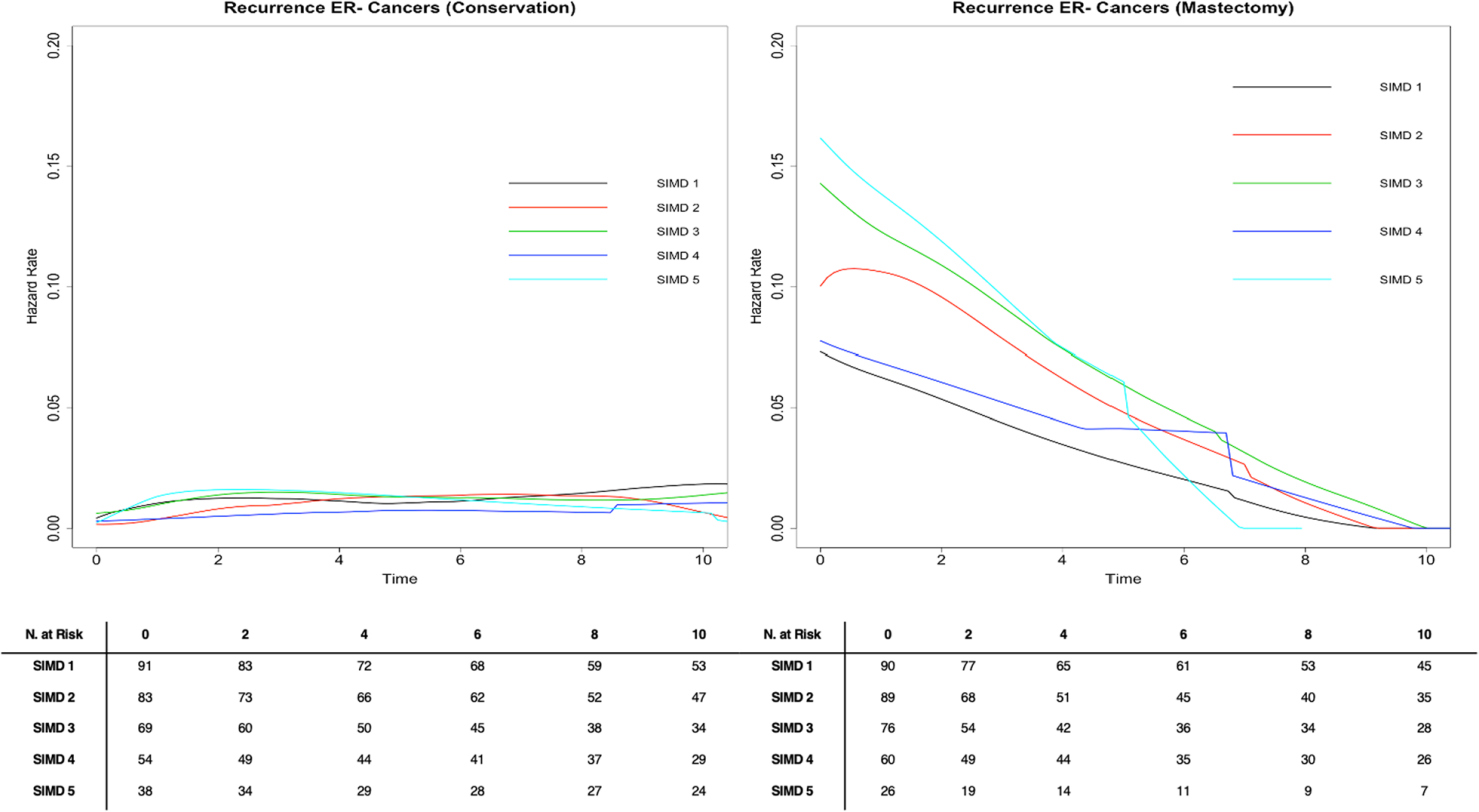
Epanechnikov kernel-smoothed hazard function curve for breast cancer recurrence stratified by surgery for 676 ER- breast cancer patients diagnosed in Scotland in 2007–2008 with 10 year follow-up data. ^1^SIMD 1 represents the most affluent area. SIMD 5 represents the most deprived area

For patients with ER- tumours who underwent breast conserving surgery, there was little notable variability in hazard rates between deprivation strata and these rates remained fairly constant over time (Fig. 5). For patients with ER- tumours who underwent mastectomy, however, the risk of IBR as shown by the hazard rate remains high in the first 2 years after diagnosis, substantially decreases over the first 3–4 years following diagnosis, and approaches the risk of recurrence of ER + tumours around 8–10 years (Fig. 5). While the rate of change in the hazard rates is similar across deprivation strata, those in the most deprived group do have a consistently higher hazard rate at each time point than those in the least deprived group (Figs. 4 and 5).

### IBR by deprivation stratum: multivariable models

In the fully-adjusted Cox proportional hazards models censored at 5 years, IBR at 5 years for patients with ER+ tumours did not vary significantly by SIMD quintile for mastectomy or breast conservation patients (Additional file 2: Table S1). In the fully-adjusted Cox proportional hazards models censored at 5 years, no patient factors or tumour characteristics significantly impacted IBR risk for patients with ER- tumours who underwent breast conserving surgery (Table 3). Patients with ER- tumours who underwent mastectomy procedures and lived in more deprived areas had a greater risk of recurrence at 5 years (Table 3). Those in SIMD 2 had an HR of 1.93 (95% CI 1.07–3.47) relative to the least deprived group (SIMD 1), those in SIMD 3 had an HR of 2.15 (1.20–3.47) at 5 years, and those in the most deprived group (SIMD 5) had a three-fold increased risk of IBR at 5 years (HR 3.03 [1.41–6.53]). Age, grade, TNM stage and receipt of chemotherapy were not associated with IBR risk for mastectomy patients. Patients with screen detected tumours had a significantly lower IBR risk at 5 years for ER- tumours (HR 0.30 [0.10–0.84]) (Table 3). Patients who underwent mastectomy and received radiation therapy had a greater than two-fold increased risk of IBR (HR: 2.59 [1.57–4.02]).

**Table 3:** Fully adjusted Cox proportional hazards model for breast cancer recurrence (IBR) among ER-negative patients censored at 5 years stratified by surgery type

Exposure^d^	ER-negative patients^c^ (n = 676) 155 IBR events	ER-negative patients with breast conservation^c^ (n = 332) 55 IBR events	ER-negative patients with mastectomy^c^ (n = 339) 100 IBR events
	HR (95% CI)		
Age < 50	(reference)	(reference)	(reference)
Age 50–70	0.82 (0.56–1.22)	0.95 (0.49–1.85)	0.70 (0.43–1.15)
Age 70 +	1.33 (0.78–2.26)	0.74 (0.23–2.40)	1.31 (0.72–2.38)
SIMD 1^a^	(reference)	(reference)	(reference)
SIMD 2	1.45 (0.91–2.31)	0.87 (0.39–1.95)	1.93 (1.07–3.47)
SIMD 3	1.88 (1.19–2.96)	1.24 (0.57–2.67)	2.15 (1.20–3.47)
SIMD 4	0.93 (0.53–1.65)	0.87 (0.35–2.20)	1.00 (0.48–2.10)
SIMD 5	2.10 (1.18–3.73)	1.24 (0.57–2.67)	3.03 (1.41–6.53)
TNM stage 1	(reference)	(reference)	(reference)
TNM stage 2/3	1.47 (0.86–2.50)	1.33 (0.66–2.66)	2.06 (0.71–5.96)
Grade 1/2^b^	(reference)	(reference)	(reference)
Grade 3	1.25 (0.77–2.03)	2.05 (0.78–5.41)	1.28 (0.72–2.28)
Not screen detected	(reference)	(reference)	(reference)
Screen detected	0.63 (0.36–1.09)	0.98 (0.46–2.08)	0.30 (0.10–0.84)
Breast-conserving surgery	(reference)	–	–
Mastectomy	2.66 (1.75–3.77)	–	–
Chemotherapy	1.24 (0.73–2.10)	0.94 (0.38–2.33)	1.49 (0.76–2.90)
Radiotherapy	1.96 (1.28–3.00)	0.17 (0.07–0.41)	2.59 (1.57–4.02)

^a^SIMD 1 represents the most affluent area. SIMD 5 represents the most deprived area

^b^Grades 1 and 2 were collapsed in the ER-model as there were very few cases in either category

^c^Analyses censored at 5 years

^d^Cancer network was included in these multivariable models

Landmark analyses could not be performed for ER- tumours in the 5–10 year follow up period as there were only 27 recurrences for the ER- cohort during this time period.

The Cox proportional hazards assumption was met for all models. Variance inflation factors (VIF) were < 4 for all models, suggesting little evidence of multicollinearity.

## Discussion

In this study of nearly 4000 Scottish women diagnosed with a primary BC from 2007 and 2008, we found no evidence of socioeconomic disparities in IBR at 5 or 10 years for patients with ER+ tumours. TNM stage and tumour grade were associated with IBR risk, as expected. Patients who survived until 5 years had no increased IBR risk between 5 and 10 years, suggesting that if patients with ER+ tumours survive for 5 years after their diagnosis, they have a lower risk of IBR. When accounting for the competing risk of death, we still did not observe socioeconomic disparities in IBR by SIMD quintile. While socioeconomic disparities in BC survival have been described in multiple Scottish studies [6, 13, 26–28], this study suggests that recurrence may not be a significant driver of this increased risk of mortality among deprived BC patients. This is consistent with a meta-analysis that found that one BC death could be avoided over the next 15 years for every four local recurrences avoided—as one death for every four recurrences suggests that recurrence is not a significant driver of mortality risk [29].

We did not observe any statistically significant differences in screen detected tumours in patients with ER+ tumours, with ~ 60% of tumours within the 50–70 age group being detected through mammographic screening for nearly all SIMD quintiles. We have previously shown that ER+ screen-detected tumour incidence rates in Scotland are lower for the most deprived compared to the least deprived [6]. One explanation could be that even if there are differences between groups, the magnitude is small and can only be observed with larger datasets; we previously observed about a 30% difference in age-standardised incidence rates for 2007 between SIMD low and high groups with the greatest difference seen in 2011 [6]. Another explanation for these discrepancies may be that this study was not a representative sample with only 25% of cases coming from WOSCAN, the largest NHS region, hence our study may not fully represent the Scottish population, with fewer deprived patients represented. Despite these limitations, it's encouraging to see within these data no evidence of an association between SIMD and IBR for ER+ tumours, which we suspect reflects the emphasis of Quality Performance Indicators [7] to ensure quality cancer care and free access to treatment through the NHS. Future population wide studies and temporal trend studies are needed to monitor outcomes.

Although based on smaller numbers, we observed patients in the most deprived quintile (SIMD 5) with ER- tumours who underwent mastectomy procedures were at a three times greater risk of IBR at 5 years when compared to the least deprived patients, and patients in intermediate SIMD quintiles (SIMD 2 and 3) had approximately a two-fold increased risk of IBR as well. Patients with ER-tumours who underwent mastectomy and received radiation therapy had greater than a two-fold increase in risk of IBR. This could potentially be due to more aggressive tumour subtypes or more advanced cancers (i.e. greater TNM stage) requiring radiation therapy when there is concern for IBR risk [30]. When taking into account the competing risk of death, a statistically significant difference in risk of IBR for deprived ER- mastectomy patients remained. Patients who undergo mastectomy may have more aggressive molecular subtypes that carry a higher risk of IBR, so while we stratified these analyses by surgical management and ER status, it is possible that some residual confounding remains [31, 32]. Patients in the breast conservation group had more Stage 1 tumours (43.7%) than Stage 2/3 (56.3%) when compared to the mastectomy group (Stage 1 = 19.0%, Stage 2/3 = 81.0%) for ER- cancers, as well as for ER+ cancers (Conservation Stage 1 = 67.9%, Stage 2/3 = 32.1%; Mastectomy Stage 1 = 26.8%, Stage 2/3 = 73.2%). These differences were statistically significant on chi square analysis (*p *< 0.001). IBR may have been more prevalent in the mastectomy group than the breast conserving group as those in the mastectomy group had more advanced cancers at diagnosis. It could also be possible that patients that warrant chemotherapy and radiotherapy in the mastectomy group have more aggressive tumours, which could contribute to the increased risk of recurrence in this group.

In a recent study of Dutch women < 40 years of age, high socioeconomic status (SES) was associated with lower recurrence risk over 10 years when compared to patients with low SES [33]. We observed a similar result in our study among patients with ER- tumours. Di Salvo et al. [34] found that deprived Italian women with ER+ tumours had a substantially higher five-year risk of recurrence than the least deprived women with ER+ tumours even after adjusting for stage and stratifying for hormone receptor status and age. In women with hormone receptor-negative cancer, SES had no significant effect on the five-year risk of recurrence [34]. While these results could potentially be due to differences in populations and differences in the healthcare systems of Scotland and Italy, further studies should investigate BC recurrence when stratifying by ER status to clarify these results.

Most studies that have evaluated socioeconomic deprivation and BC screening have focused on its association overall and not by subtypes. While data have shown that higher deprivation groups are less likely to attend screening overall, and increasing incidence has primarily been observed for ER+ tumours—limited data have evaluated this for ER- tumours [6, 35]. A greater proportion of those aged 50–70 in the most deprived group had screen-detected ER- tumours when compared to those aged 50–70 in less deprived categories. Possible explanations for this difference in the proportion of screen-detected tumours by SIMD quintile include more deprived patients not seeking clinic referral, and that the NHS Breast Cancer Screening Programme provides more deprived patients with an avenue to interact with the NHS and engage with their breast health. We know from Public Health Scotland data that there are slightly lower participation rates by SIMD (59.5% uptake in 2016–2019 in the most deprived areas of Scotland compared to 79.7% in the least deprived areas) [36]. Less deprived patients may be more likely to identify symptoms of early or recurrent BC on their own and may have more time, flexibility, and persistence that allow them to present to their GP with concerns, which would result in more tumours detected by proactive self-referral than screen-detection [37, 38]. The Detect Cancer Early Programme was formally implemented by the Scottish Government in 2012 (approximately 5 years after the patients in this cohort were diagnosed with BC), so future studies should investigate the role that this programme has played in BC recurrence and survival in Scotland [39].

For patients with ER- tumours who underwent mastectomy, the risk of IBR as shown by the hazard rate remained high in the first 2 years after diagnosis, substantially decreased over the first 3–4 years following diagnosis, and approached the risk of IBR of ER+ tumours around 8–10 years. This could highlight a need for closer follow up in the first 2–4 years following diagnosis for patients with ER- tumours, especially those who underwent mastectomy. Perhaps this closer follow-up may help mitigate some of the observed disparities in recurrence by deprivation for patients with ER- cancers. The differences observed in hazard rates for ER+ and ER- tumours may also suggest different behaviour and aetiology of ER+ and ER- BCs. One limitation of these models at later time points (8–10 years) is the smaller number at risk, so these estimates may represent a true effect or may be an artefact.

This study has several strengths as to our knowledge it is the first study in the United Kingdom to investigate BC recurrence and survival by deprivation and ER subtypes utilising high-quality data from the Scottish Cancer Audit with linkage to mortality records. As this is an observational study, the validity of our findings is subject to bias and potential confounders. Our multivariable analyses controlled for two major potential confounders, ER status and breast surgery, but we were unable to adjust for other risk factors for recurrence such as HER2 status and trastuzumab therapy as HER2 was not routinely reported to cancer networks in Scotland at the time of this study, and trastuzumab was being introduced as routine therapy on NHS Scotland around the same time. This is a limitation of this study, as HER2 has been shown to be associated with BC mortality and SIMD [6]. This study may serve as a reference point for disparities in BC recurrence prior to provision of trastuzumab in the NHS and prior to changes in surgical management of BC over the past 15 years. Future studies using recent data should investigate the impact of the expanded access to these treatments on disparities in BC recurrence.

Type of breast surgery was found to vary by cancer network, suggesting that access to hospitals and rural location may impact cancer treatment. Barriers to radiation therapy may be greater for patients in more remote locations, which may have impacted patient and surgeon choices when considering breast conserving surgery versus mastectomy. While cancer network was included in the adjusted analyses, there may be residual confounding present as treatment has been shown to vary by cancer network in previous Scottish studies [40]. This cancer audit dataset is missing data from multiple health boards, most notably the Greater Glasgow area, which make the results not generalizable to this area. Missing HER2 data and TNM stage data are also a limitation. Furthermore, lack of information on comorbidities, smoking status, alcohol use, and BMI can also be considered a limitation of this study given that these factors may impact a person’s risk of recurrence [33, 41]. Age was not available as a continuous variable because of patient confidentiality, so there is a possibility of residual confounding by age in these analyses. There is potential for misclassification for recurrence as well, as it may be difficult to distinguish between recurrence and second primary tumours. SIMD is an area-based measure of deprivation, so it has been shown to misclassify individuals’ SES [42]. The potential for misclassification is greatest among rural areas, as the ‘access’ domain does not capture unique characteristics of rural areas, such as cost and frequency of public transport [43].

## Conclusions

IBR is not a major contributor to mortality differences by SIMD for the majority of BC patients. The lack of inequities in IBR is likely due to standardised treatment protocols and access to healthcare. The association with socioeconomic deprivation and recurrence for ER- tumours requires further study.

### Supplementary Information

**Additional file 1.** Supplemental Methods including data and cohort definitions, missing data and statistical analysis.

**Additional file 2: Table S1.** Fully Adjusted Cox Proportional Hazards Model for ER+ Breast Cancer Recurrence Censored at 5 Years Stratified by Surgery.

## Data Availability

The datasets generated are from the NHS and are not publicly available due to privacy regulations, but authors will aim to work with others on reasonable request.

## References

[1] Sung H, Ferlay J, Siegel RL, Laversanne M, Soerjomataram I, Jemal A, Bray F (2021). Global cancer statistics 2020: GLOBOCAN estimates of incidence and mortality worldwide for 36 cancers in 185 countries. CA Cancer J Clin.

[2] Scottish Public Health Observatory. Breast cancer: Scottish data [Online]. ISD Scotland. 2020. Accessed 16 May 2020 from https://www.scotpho.org.uk/health-wellbeing-and-disease/cancer-breast/data/scottish/

[3] Polyak K (2011). Heterogeneity in breast cancer. J Clin Invest.

[4] Gaudet MM, Press MF, Haile RW, Lynch CF, Glaser SL, Schildkraut J (2011). Risk factors by molecular subtypes of breast cancer across a population- based study of women 56 years or younger. Breast Cancer Res Treat.

[5] Tamimi RM, Colditz GA, Hazra A, Baer HJ, Hankinson SE, Rosner B (2012). Traditional breast cancer risk factors in relation to molecular subtypes of breast cancer. Breast Cancer Res Treat.

[6] Mesa-Eguiagaray I, Wild SH, Bird SM, Williams LJ, Brewster DH, Hall PS, Figueroa JD (2022). Breast cancer incidence and survival in Scotland by socio-economic deprivation and tumour subtype. Breast Cancer Res Treat.

[7] Healthcare Improvement Scotland. Cancer quality performance indicators (QPIs). 2023. Accessed 23 Feb 2023 from https://www.healthcareimprovementscotland.org/our_work/cancer_care_improvement/cancer_qpis.aspx

[8] Mullooly M (2017). Divergent oestrogen receptor-specifc breast cancer trends in Ireland (2004–2013): amassing data from independent Western populations provide etiologic clues. Eur J Cancer.

[9] Engstrøm MJ (2013). Molecular subtypes, histopathological grade and survival in a historic cohort of breast cancer patients. Breast Cancer Res Treat.

[10] Fallahpour S (2017). Breast cancer survival by molecular subtype: a population-based analysis of cancer registry data. CMAJ Open.

[11] Howlader N (2018). Difefrences in breast cancer survival by molecular subtypes in the United States. Cancer Epidemiol Biomark Prev.

[12] Anderson WF, Katki HA, Rosenberg PS (2011). Incidence of breast cancer in the United States: current and future trends. J Natl Cancer Inst.

[13] Johansson ALV (2019). Breast cancer−specifc survival by clinical subtype after 7 years follow-up of young and elderly women in a nationwide cohort. Int J Cancer.

[14] Macmillan Cancer Support. Deprivation and cancer survival in Scotland: technical report. 2017. Available https://www.macmillan.org.uk/_images/ISD%20Macmillan%20Deprivation%20Survival%20Technical%20Report_FINAL_tcm9-308832.pdf

[15] Jang B-S, Chang JH (2019). Socioeconomic status and survival outcomes in elderly cancer patients: a national health insurance service-elderly sample cohort study. Cancer Med.

[16] Pruitt SL (2009). Association of area socioeconomic status and breast, cervical, and colorectal cancer screening: a systematic review. Cancer Epidemiol Biomark Prev.

[17] Schrijvers CT (1995). Deprivation and survival from breast cancer. Br J Cancer.

[18] Woods LM (2016). Impact of deprivation on breast cancer survival among women eligible for mammographic screening in the West Midlands (UK) and New South Wales (Australia): women diagnosed 1997–2006. Int J Cancer.

[19] Tanis E, Van De Velde CJH, Bartelink H, van de Vijver MJ, Putter H, van der Hage JA (2012). Locoregional recurrence after breast-conserving therapy remains an independent prognostic factor even after an event free interval of 10 years in early stage breast cancer. Eur J Cancer.

[20] Lee JS, Kim SI, Park HS, Lee JS, Park S, Park BW (2011). The impact of local and regional recurrence on distant metastasis and survival in patients treated with breast conservation therapy. J Breast Cancer.

[21] SCOTTISH GOVERNMENT. 2020. Scottish index of multiple deprivation 2020: introduction.

[22] Public Health Intelligence. Deprivation guidance for analysts. In: Scotland, N. (editor). 2017.

[23] Jatoi I, Pinsky PF (2021). Breast cancer screening trials: endpoints and overdiagnosis. JNCI J Natl Cancer Inst.

[24] Public Health Scotland (2022). Breast screening. Accessed 1 Jan 2023 from https://www.healthscotland.scot/health-topics/screening/breast-screening

[25] Amin MB (2017). The eighth edition AJCC cancer staging manual: continuing to build a bridge from a population-based to a more “personalized” approach to cancer staging. CA Cancer J Clin.

[26] Carnon AG, Ssemwogerere A, Lamont D, Hole D, Mallon E, George W, Gillis CR (1994). Relation between socioeconomic deprivation and pathological prognostic factors in women with breast-cancer. Br Med J.

[27] Twelves CJ, Thomson CS, Gould A, Dewar JA (1998). Variation in the survival of women with breast cancer in Scotland. The Scottish breast cancer focus group and the Scottish cancer therapy network. Br J Cancer.

[28] Shack L, Rachet B, Brewster D, Coleman M (2007). Socioeconomic inequalities in cancer survival in Scotland 1986–2000. Br J Cancer.

[29] Early Breast Cancer Trialists' Collaborative Group (2005). Effects of radiotherapy and of differences in the extent of surgery for early breast cancer on local recurrence and 15-year survival: an overview of the randomised trials. Lancet.

[30] Olivotto IA, Truong PT, Chua B (2004). Postmastectomy radiation therapy: who needs it?. J Clin Oncol.

[31] Tseng YD, Uno H, Hughes ME, Niland JC, Wong YN, Theriault R, Blitzblau RC, Moy B, Breslin T, Edge SB, Hassett MJ (2015). Biological subtype predicts risk of locoregional recurrence after mastectomy and impact of postmastectomy radiation in a large national database. Int J Radiat Oncol Biol Phys.

[32] Hess SobK, Gentleman RpbR. Muhaz: hazard function estimation in survival analysis. 2021. R package version 1.2.6.4, https://CRAN.R-project.org/package=muhaz

[33] van Maaren MC, Rachet B, Sonke GS, Mauguen A, Rondeau V, Siesling S, Belot A (2022). Socioeconomic status and its relation with breast cancer recurrence and survival in young women in the Netherlands. Cancer Epidemiol.

[34] di Salvo F, Caranci N, Spadea T, Zengarini N, Minicozzi P, Amash H, Fusco M, Stracci F, Falcini F, Cirilli C (2017). Socioeconomic deprivation worsens the outcomes of Italian women with hormone receptor-positive breast cancer and decreases the possibility of receiving standard care. Oncotarget.

[35] Smith D, Thomson K, Bambra C, Todd A (2019). The breast cancer paradox: a systematic review of the association between area-level deprivation and breast cancer screening uptake in Europe. Cancer Epidemiol.

[36] Public Health Scotland. Scottish breast screening pogramme statistics 2018/2019. 2019. https://www.isdscotland.org/Health-Topics/Cancer/Publications/2017-04-25/2017-04-25-SBSP-CancER−Report.pdf

[37] Hvidberg L, Pedersen AF, Wulff CN, Vedsted P (2014). Cancer awareness and socio-economic position: results from a population-based study in Denmark. BMC Cancer.

[38] Whitaker KL, Scott SE, Wardle J (2015). Applying symptom appraisal models to understand sociodemographic differences in responses to possible cancer symptoms: a research agenda. Br J Cancer.

[39] Public Health Scotland (2023). Detect cancer early programme. Accessed 4 Feb 2023 from https://www.isdscotland.org/Health-Topics/Cancer/Detect-Cancer-Early/

[40] Gray E, Figueroa JD, Oikonomidou O, MacPherson I, Urquhart G, Cameron DA, Hall PS (2021). Variation in chemotherapy prescribing rates and mortality in early breast cancer over two decades: a national data linkage study. ESMO Open.

[41] Azrad M, Demark-Wahnefried W (2014). The association between adiposity and breast cancer recurrence and survival: a review of the recent literature. Curr Nutr Rep.

[42] Tunstall R, Lupton R (2003). Is targeting deprived areas an efective means to reach poor people? An assessment of one rationale for area-based funding programmes, CASE/70.

[43] Robson B (2001). Deprivation in London: an alternative to IMD 2000.

