# Supplementary material for: Evaluation of the association of area-level socioeconomic deprivation and breast cancer recurrence by oestrogen receptor subtypes in Scotland
Source: Breast Cancer Res. 2023 Oct 3;25:106. doi: 10.1186/s13058-023-01704-6 (PMC10546786; doi:10.1186/s13058-023-01704-6)
Supplement: Supplementary file 1 — Additional file 1. Supplemental Methods including data and cohort definitions, missing data and statistical analysis. [file 13058_2023_1704_MOESM1_ESM.docx]

**SUPPLEMENTAL METHODS**

**Data and Cohort Definition**

*SIMD*

The SIMD uses postcodes to determine whether a person lives in a deprived area. SIMD is often separated into quintiles for ease of interpretation [31].

*Mode of detection*

In Scotland, women aged 50-70 are invited for screening every three years [34]. While there are some Scottish women who have screening mammograms before age 50 or after age 70, this is not a large population [34].

*Cancer Network*

WoSCAN sites included Ayrshire and Lanarkshire, NOSCAN sites included Grampian and Highland and SCAN included Borders, Fife and Lothians.

*TNM Stage*

Using tumour size and the number of positive nodes, a TNM stage variable was derived and categorised as I, II, III, and IV following the American Joint Committee on Cancer (AJCC) 8^th^ Edition Cancer Staging manual [36].

*Surgery*

Breast conservation was defined as removal of tumour with surrounding normal tissue without the removal of the whole breast. Wide local excision, segmentectomy and quadrantectomy are included in the breast conservation group. Mastectomy included removal of the breast/axillary contents and perhaps pectoral muscles.

Deaths

Data on vital status was collected from Scottish Morbidity Records, specifically National Records of Scotland Deaths data [38].

*Recurrence*

The endpoint of BC recurrence was based on local investigator review and not centrally assessed. Ipsilateral breast recurrence (IBR) was defined using the criteria provided in Standardized Definitions for Efficacy End Points (STEEP) in Adjuvant Cancer Clinical Trials [53].

**Missing Data**

Approximately 36% of cases were missing HER2 status, and this missingness was found to be associated with cancer network. HER2 status was not routinely reported for all cancer networks until 2010, and at the time of diagnosis for this study cohort, trastuzumab was not uniformly available across Scottish cancer networks. HER2 status, therefore, was excluded from the final multivariable models. TNM stage was missing for 30.2% of cases which was likely to do with the proportion of cases missing N stage (25.5%) and the proportion of cases missing T stage (26.3%). Missingness for TNM status was also associated with SIMD, with greater proportion of cases in SIMD1 missing TNM status compared to those in SIMD5 (data not shown). To ensure adequate statistical power, “missing” was included as an additional level of the HER2 status variable and the TNM status variable. Missing from WoSCAN is Forth Valley and Greater Glasgow, missing from NoSCAN is Orkney and Western Isles, and missing from SCAN is Dumfries and Galloway.

**Statistical Analysis**

*Stratification by Surgery*

Patients who undergo mastectomy may have more aggressive molecular subtypes that carry a higher risk of recurrence [39,40]. Patients who undergo breast conservation surgery also have remaining breast tissue in which a cancer could potentially recur.

*Model Diagnostics*

We tested for violations of the proportional hazards function using Schoenfeld residuals. Final multivariate Cox models included covariates that were associated with recurrence at the p<0.1 level. Variable Inflation Factors (VIF) were calculated for these multivariable models to assess for multicollinearity.
