# Supplementary material for: Evaluation of the association of area-level socioeconomic deprivation and breast cancer recurrence by oestrogen receptor subtypes in Scotland
Source: Breast Cancer Res. 2023 Oct 3;25:106. doi: 10.1186/s13058-023-01704-6 (PMC10546786; doi:10.1186/s13058-023-01704-6)
Supplement: Supplementary file 2 — Additional file 2: Table S1. Fully Adjusted Cox Proportional Hazards Model for ER+ Breast Cancer Recurrence Censored at 5 Years Stratified by Surgery. [file 13058_2023_1704_MOESM2_ESM.docx]

**SUPPLEMENTAL TABLE 1. Fully Adjusted Cox Proportional Hazards Model for Breast Cancer Recurrence (IBR) Censored at 5 Years Stratified by Surgery Type.**

| **Exposure** | **ER+ Breast Cancer Patients^2^**  **(n=2819)**  **247 IBR events** | **ER+ Patients with Conservation^2^**  **(n=1721)**  **96 IBR events** | **ER+ Patients with Mastectomy^2^**  **(n=1084)**  **151 IBR events** | **ER+ Patients with Breast Conservation From 5-10 Years**  **(n=1500)**  **76 IBR events** | **ER+ Patients with Mastectomy From 5-10 Years**  **(n=854)**  **78 IBR events** |
| --- | --- | --- | --- | --- | --- |
|  | **HR**  **(95% CI)** |  |  |  |  |
| **Age <50** | (reference) | (reference) | (reference) | (reference) | (reference) |
| **Age 50-70** | 0.78  (0.56-1.07) | 0.50  (0.41-1.22) | 0.77  (0.52-1.15) | 1.74  (0.41-1.22) | 1.15  (0.63-2.09) |
| **Age 70+** | 0.84  (0.55-1.28) | 0.51  (0.25-1.05) | 1.03  (0.59-1.79) | 2.32  (1.00-5.40) | 1.95  (0.90-4.22) |
| **SIMD 1^1^** | (reference) | (reference) | (reference) | (reference) | (reference) |
| **SIMD 2** | 0.90  (0.63-1.30) | 0.70  (0.38-1.29) | 0.98  (0.62-1.55) | 1.68  (0.96-2.93) | 0.65  (0.33-1.30) |
| **SIMD 3** | 1.13  (0.79-1.62) | 1.39  (0.82-2.37) | 0.91  (0.56-1.58) | 0.61  (0.28-1.33) | 1.09  (0.59-2.03) |
| **SIMD 4** | 0.76  (0.51-1.15) | 0.43  (0.19-0.95) | 0.90  (0.55-1.47) | 0.75  (0.34-1.62) | 0.75  (0.37-1.50) |
| **SIMD 5** | 1.16  (0.76-1.78) | 1.28  (0.57-2.45) | 1.15  (0.65-2.03) | 0.93  (0.40-2.20) | 0.75  (0.31-1.79) |
| **TNM Stage 1** | (reference) | (reference) | (reference) | (reference) | (reference) |
| **TNM Stage 2/3** | 1.87  (1.31-2.67) | 2.62  (1.62-4.23) | 1.46  (0.84-2.55) | 1.85  (1.06-3.23) | 0.61  (0.32-1.16) |
| **Grade 1** | (reference) | (reference) | (reference) | (reference) | (reference) |
| **Grade 2** | 1.76  (1.00-3.10) | 2.79  (1.25-6.23) | 1.20  (0.54-2.66) | 0.85  (0.44-1.65) | 1.15  (0.48-2.77) |
| **Grade 3** | 3.00  (1.67-5.41) | 3.68  (1.53-8.84) | 2.40  (1.08-5.33) | 0.76  (0.34-1.67) | 2.01  (0.80-5.07) |
| **HER2-** | (reference) | (reference) | (reference) | (reference) | (reference) |
| **HER2+** | 0.83  (0.55-1.25) | 0.45  (0.18-1.00) | 1.15  (0.71-1.86) | 0.78  (0.32-1.93) | 0.46  (0.18-1.19) |
| **Not Screen Detected** | (reference) | (reference) | (reference) | (reference) | (reference) |
| **Screen Detected** | 0.52  (0.36-0.77) | 0.66  (0.39-1.13) | 0.44  (0.24-0.79) | 0.39  (0.22-0.68) | 0.82  (0.45-1.50) |
| **Breast-Conserving Surgery** | (reference) | ---- | ---- | ---- | ---- |
| **Mastectomy** | 2.08  (1.52-2.84) | ---- | ---- | ---- | ---- |
| **Chemotherapy** | 1.05  (0.73-1.51) | 0.94  (0.53-1.64) | 1.14  (0.69-1.89) | 2.55  (1.38-4.72) | 1.16  (0.61-2.21) |
| **Radiotherapy** | 1.35  (0.98 – 1.87) | 0.59  (0.29-1.22) | 1.55  (1.07-2.25) | 0.85  (0.34-2.02) | 1.57  (0.94-2.64) |

^1^ SIMD 1 represents the most affluent area. SIMD 5 represents the most deprived area.

^2^Analyses censored at five years.
